# Heart Failure Drug Class Effects on 30-Day Readmission Rates in Patients with Heart Failure with Preserved Ejection Fraction: A Retrospective Single Center Study

**DOI:** 10.3390/medicines7050030

**Published:** 2020-05-20

**Authors:** Priyanka Parajuli, Odalys Estefania Lara-Garcia, Manjari Rani Regmi, Warren Skoza, Mukul Bhattarai, Abhishek Kulkarni, Robert Leonard Robinson

**Affiliations:** 1 Department of Internal Medicine, Southern Illinois University School of Medicine, Springfield, IL 62702, USA; olaragarcia85@siumed.edu (O.E.L.-G.); mregmi81@siumed.edu (M.R.R.); wskoza53@siumed.edu (W.S.); rrobinson@siumed.edu (R.L.R.); 2 Division of Cardiology, Department of Internal Medicine, Southern Illinois University School of Medicine, Springfield, IL 62702, USA; mbhattarai28@siumed.edu (M.B.); akulkarni52@siumed.edu (A.K.)

**Keywords:** heart failure, heart failure with preserved ejection fraction, readmission, 30-day readmission, antihypertensive medication

## Abstract

**Background:** The pharmacologic management of heart failure with preserved ejection fraction (HFpEF) involves far fewer options with demonstrated additional benefit. Therefore, we examined the effect of combination of multiple classes of HF medication in the 30-day hospital readmission in patients with HFpEF. **Methods:** All adult patients discharged with a diagnosis of HFpEF and a left ventricular ejection fraction (LVEF) of ≥ 50% reported during the admission or within the previous six months from our institution were retrospectively studied for a 30-day hospital readmission risk. Individual as well as combination drug therapy at the time of hospital discharge were evaluated using Pearson chi^2^ test and multivariate logistic regression. **Results:** The overall 30-day readmission rate in this HFpEF cohort of 445 discharges was 29%. Therapy with loop diuretics (*p* = 0.011), loop diuretics and angiotensin receptor blocker (*p* = 0.043) and loop diuretics and beta blockers (*p* = 0.049) were associated with a lower risk of 30-day hospital readmission. Multivariate logistic regression revealed only loop diuretics to be associated with a lower risk of hospital readmission in patients with HFpEF (OR 0.59; 95% CI, 0.39-0.90; *p* = 0.013). **Conclusions:** Our study revealed that loop diuretics at discharge decreases early readmission in patients with HFpEF. Further, our study highlights the implication of a lack of guidelines and treatment challenges in HFpEF patients and emphasizes the importance of a conservative approach in preventing early readmission in patients with HFpEF.

## 1. Introduction

Heart failure (HF) is a clinical syndrome affecting nearly six million Americans [1]. It is a heterogenous condition with various mechanisms of disease formation, and as such has several important clinical distinctions. Heart failure with preserved ejection fraction (HFpEF) is defined as a left ventricular ejection fraction (LVEF) greater than 50%, with the presence of a normal left ventricular end-diastolic volume (LVEDV) [2]. This clinical entity becomes increasingly prevalent with age, and is more common in women regardless of age [3]. The six-month hospitalization rate in patients with HFpEF is approximately 50%, which is comparable to that of patients with HFrEF. Furthermore, the hospitalization rates of patients with HFpEF are more than double those of matched cohorts with relevant comorbidities including hypertension, coronary artery disease, or diabetes mellitus but without heart failure [4]. Hospitalizations related to heart failure are an enormous burden on the United States healthcare system. In 2014, approximately one million hospitalizations included HF as a primary diagnosis or underlying cause of death. Each primary HF hospitalization costs an average of 11,500 USD, combining for a total estimated annual cost of 11 billion USD. Additionally, over three million hospitalizations included HF as a comorbid diagnosis [5]. With the expansion of value-based purchasing programs such as that utilized by the Centers for Medicare & Medicaid Services (CMS), health care systems are increasingly incentivized to reduce the number of patients that return for care within a certain time period of treatment. For instance, the Hospital Readmissions Reduction Program (HRRP) penalizes hospitals with excessive 30-day readmission rates [6]. HF admissions are an area of particular interest due to the high disease prevalence and costs for both index and repeat hospitalizations. While incentivization has successfully reduced readmission rates in the past decade for conditions such as HF, the rate of reduction has slowed in recent years, and adverse effects such as increased mortality have been documented [7]. This highlights the necessity for improved medical management of HFpEF, both in terms of optimization of currently available therapies and the development of novel therapies targeting specific cellular mechanisms. Several large randomized controlled trials have evaluated the effect on mortality of medical therapy in patients with HFpEF and the fact remains that no single treatment modality for HFpEF has been convincingly shown to improve mortality. Therefore, given the need for improved medical therapy of HFpEF and the significant health care burden of hospital readmissions, this study aims to examine one aspect of HFpEF therapy by evaluating the 30-day readmission rate of patients with HFpEF treated with combination of various classes of antihypertensive agents at a single academic center.

## 2. Methods

**Figure 1:**
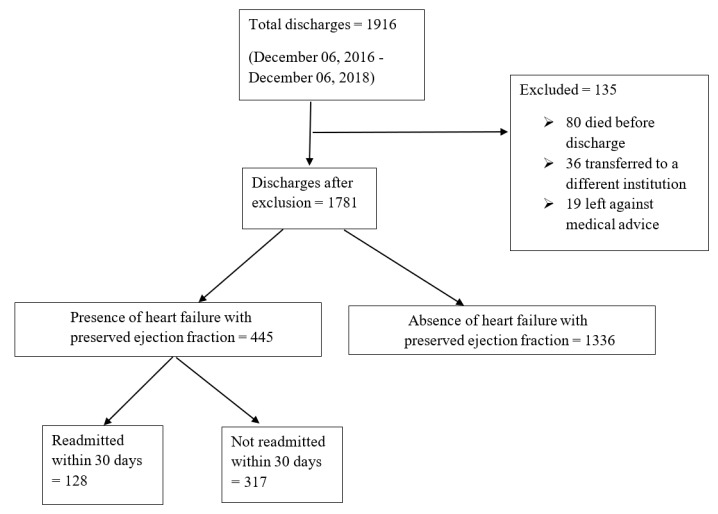
A diagrammatic representation of the flow of the study and distribution of total study size.

All adult patients discharged from the Southern Illinois University School of Medicine Hospitalist service with an International Classification of Disease (ICD), 10th Revision, Clinical Modification diagnosis of diastolic heart failure either as a primary or secondary diagnosis and an echocardiographic finding of LVEF ≥ 50% reported within the previous six months of index hospitalization were studied for a 30-day readmission risk. The discharge date of the index hospitalization was considered day zero. Patients involved in the study were managed at Memorial Medical Center (MMC) from 6 December 2016 to 6 December 2018. MMC is a 507-bed not-for-profit, university-affiliated tertiary care center in Springfield, Illinois. Patients that transferred to a different institution for any reason, left the hospital against medical advice or died during the hospitalization were excluded from the study. Of 1916 patients discharged from the hospital, a cohort of 445 discharges met the inclusion criteria and was investigated to understand the impact of various combination of cardioprotective drug therapy on the rate of hospital readmissions in patients with HFpEF. Of these discharges, 128 (29%) were readmitted to our institution within 30 days. Figure 1 illustrates a diagrammatic representation of the flow of the study.

Patients’ characteristics including age, and drug therapy at the time of discharge leading to hospital readmission was derived from medical records. Preexisting clinical condition was defined using ICD-9 and ICD-10 codes and was also derived from patient’s medical record (Table 1). The Springfield Committee for Research Involving Human Subjects provided ethical oversight for the research and reviewed the protocol (Reference Number 016815). The review determined this project did not fall under the purview of the IRB as research involving human subjects according to 45 CFR 46.101 and 45 CFR 46.102.

As a de-identified study with information from only a single hospital, data on readmissions at other hospitals, death outside the hospital, and therapeutic adherence are not available for study. The readmission data from the hospital indicates if the patient was readmitted, cause of the readmission is not linked to the data from the index hospital stay.

Drug therapy at the time of discharge with beta blockers (BB), ACE inhibitors (ACEI), angiotensin receptor blockers (ARB), loop diuretics, and spironolactone were investigated. Further, combination therapy at the time of discharge with Loop + BB, Loop + ACEI, Loop + ARB, Loop + spironolactone, Loop + BB+ ACEI, Loop + BB + ARB, Loop + ACEI + spironolactone, Loop + ARB+ Spironolactone, Loop + BB + spironolactone, Loop + BB + ACEI + spironolactone, and loop + BB + ARB + spironolactone were evaluated (Table 2). Atenolol, bisoprolol, carvedilol, labetalol, metoprolol succinate and metoprolol tartrate were BB used in the drug combination. Similarly, ARB therapy included losartan and valsartan, and ACEI therapy included benazepril, enalapril, and lisinopril. Loop diuretics therapy comprised bumetanide, torsemide and furosemide. 

Qualitative variables were compared using Pearson chi2 or Fisher’s exact test. Quantitative variables were compared using the non-parametric Mann–Whitney U test and reported as mean ± standard deviation. Variables from univariate analysis with a *p*-value of ≤ 0.10 were further evaluated using multivariate logistic regression analysis. Item included in regression analysis included Loop, Loop + ARB, Loop + BB, hypertension, atrial fibrillation, and body mass index (BMI). The odds ratio was used to measure the potential risk, along with a 95% confidence interval (CI). Variables found to be significant in the multivariate analysis were used for Kaplan–Meier analysis. The log rank statistic was calculated for each comparison. Statistical analyses were performed using SPSS version 25 (SPSS Inc., Chicago, IL, USA). A *p*-value of 0.05 was chosen for statistical significance. 

## 3. Results

Our final cohort included 445 discharges from 2016 to 2018. The two groups of HFpEF without 30-day readmission (not-readmitted group) and HFpEF with a 30-day readmission (readmitted group) included 317 and 128 patients, respectively. Overall, the baseline characteristics of patients were comparable between the two study groups including age, LVEF, and medical co-morbidities. 

**Figure 2:**
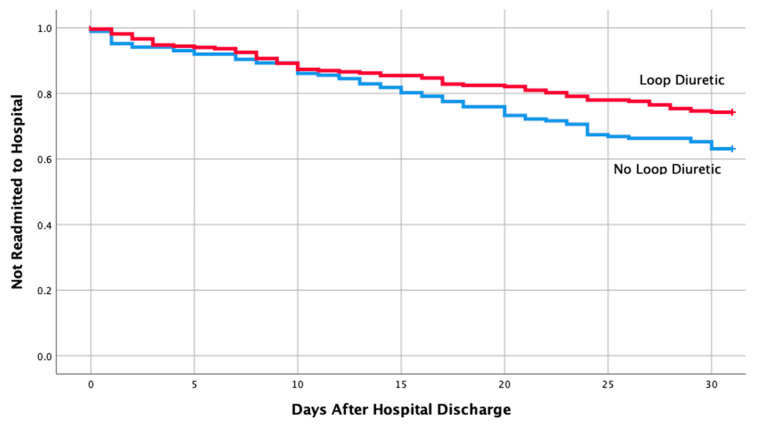
Readmission free survival for patients treated with loop diuretics.

**Figure 3:**
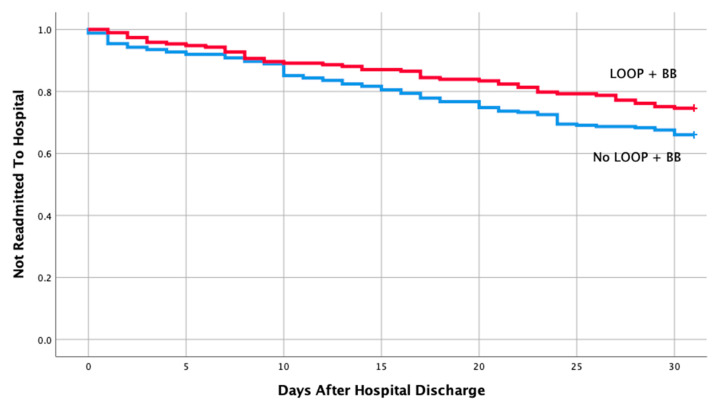
Readmission free survival for patients treated with loop + Beta blocker.

**Figure 4:**
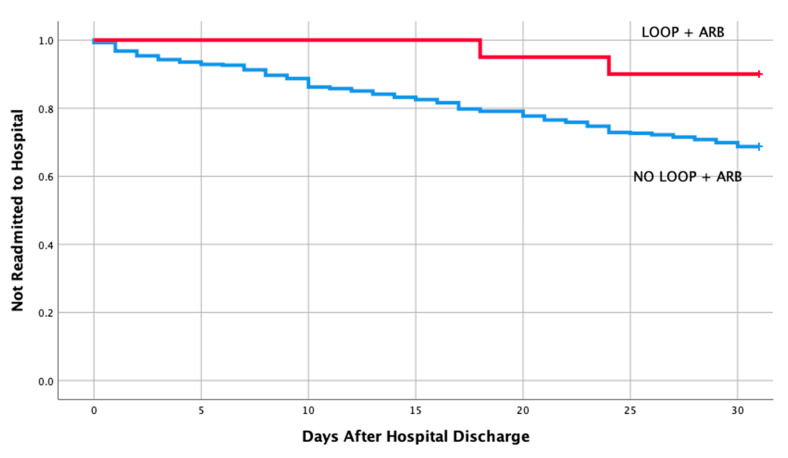
Readmission free survival for patients treated with loop + angiotensin receptor blocker.

Table 1 shows the baseline characteristics and comorbidities for the two groups which included the echocardiographic data on LVEF, pulmonary artery pressure (PAP), and ratio between early mitral inflow velocity and mitral annular early diastolic velocity (***E***/***e***’ ratio). The mean EF in both groups were 57% and 58%, respectively, in readmitted and non-readmitted group. Table 2 shows individual discharge medication class and combination of various medication classes by readmission status. Therapy comprising of loop diuretics (*p* = 0.011), loop diuretics and angiotensin receptor blocker (*p* = 0.043), and loop diuretics and beta blockers (*p* = 0.049) upon discharge were associated with a lower risk of 30-day hospital readmission. Discharge medication including ACEI, ARB, BB and spironolactone alone had no impact on a 30-day readmission (*p* = 0.106, 0.740, 0.22, 0.829, respectively). Furthermore, discharge medication including other combination of various medication classes listed in Table 2 did not have any impact on a 30-day readmission. Moreover, multivariate logistic regression of potential risk factor for 30-day readmission revealed only loop diuretics to be associated with a lower risk of hospital readmission in patients with HFpEF (OR 0.59; 95% CI, 0.39-0.90; *p* = 0.013). Kaplan–Meier analysis showed statistically significant differences in readmission free survival for patients treated with loop diuretics (Figure 2, *p* = 0.013) and Loop + BB (Figure 3, *p* = 0.048). No significant difference was seen with Loop + ARB therapy (Figure 4, *p* = 0.051).

## 4. Discussion

Our study showed that diuretic therapy is an essential component of most therapeutic regimens for HFpEF and plays an especially critical role in decompensated and volume overloaded patients. Loop diuretics are used frequently over other classes due to their short-acting onset of action and their higher efficacy in producing natriuresis, characteristics that are critical in the acute setting [8]. Loop diuretics have not shown a reduced mortality in either forms of heart failure. In our study, a reduction in hospital readmission has been noted in the use of diuretic therapy and patients with HFpEF using multivariate and Kaplan–Meier analysis. The findings of our study align with the findings of CHAMPION trial, a randomized clinical trial including patients with both HFrEF and HFpEF that demonstrated a significant clinical impact in reduction of hospitalization rates more pronounced in the HFpEF treatment group with the use of loop diuretic therapy. HF hospitalization rate for patients with both HFpEF and HFrEF in six months was static and significantly lower in the treatment group compared to the control group [9]. In our study, the readmission rate is quite high at 27%, but is comparable with previous results at this center ranging from 12% to 27% [10,11,12,13]. Local factors that may contribute to this higher than expected rate of readmission can be attributed to a lack of a multidisciplinary heart failure management clinic and a high proportion of patients with negative social determinants of health such as poverty, poor healthcare access, and rural residence. Our study findings, however, are in contrast to the findings in patient with HFrEF in which high dose diuretics, along with resistance to diuretic agents, poses a risk of worsening renal function during and after hospital admission and entails a poor clinical outcome in patients with HFrEF [14]. Further, Okabe et al. showed increased cardiovascular mortality in Japanese patients with HF regardless of LVEF discharged with a high dose (>40 mg/day) of loop diuretics [15]. Similarly, another study by Mecklai et al. showed increased all-cause mortality and increased rehospitalization in the HFrEF group treated with high-dose loop diuretics (>160 mg/day) compared to patients treated with a lower dose (<160 mg/day). However, it was also noted that patients requiring higher dose diuretics for decongestion were clinically more ill and when adjusted for covariates for disease severity the dose dependent effect of diuretic did not increase 30-day risk of combined adverse outcome [16]. A plausible explanation to patients with HFpEF experiencing better outcome with loop diuretics could be that hypertension is one of the most commonly associated co-morbidities in HFpEF patients. Since diuretics help lower the blood pressure as well as decongestion, it might better suit patients with HFpEF. A future study evaluating dose-dependent loop diuretics’ effect on a 30-day hospital readmission and long-term outcome in patients with HFpEF is bound to provide more insight to our current knowledge. 

Beta-blocker therapy (BBT) is known to have mortality benefits in HFrEF. However, there has not been any substantial evidence on BBT for reducing mortality or hospitalizations in HFpEF [17]. A meta-analysis consisting of 15 observational studies revealed decreased all-cause mortality with BBT in HFpEF. However, the same metanalysis revealed BBT did not lower hospitalization in patients with HFpEF [17]. Similarly, another metanalysis by Feng et al. evaluated the role of individual class of HF medications at discharge and demonstrated renin-angiotensin system inhibitors (RASI), and BB to reduce the all-cause mortality in patients with HFpEF but not HF readmission [18]. Two randomized control trials, SENIORS and J-DHF, revealed neither mortality nor hospitalization benefit with BBT in patients with HFpEF [19,20]. Our retrospective study correlated with findings of the observational studies included in the meta-analysis. Patients who were on various BBT, including the ones with mortality benefits (bisoprolol, carvedilol and metoprolol succinate), had no impact on patient readmission in HFpEF. Further, BBT when combined with other class of medications used to treat heart failure, including ACEI and ARB, did not prevent or lower readmission in patients with HFpEF. 

Similar to BBT, ACEI have proven mortality and hospitalization benefits in HFrEF but not in HFpEF [21]. A large randomized controlled trial with ACEI, PEP-CHF study, with perindopril, showed no mortality benefit of ACEI in HFpEF. The study did, however, reveal fewer hospitalizations in the first year of therapy [22]. Similarly, a meta-analysis by Khan et al., consisting of 13 retrospective studies and pooled analysis of seven randomized control trials for ACEI and ARBs, showed no benefits in mortality in patients with HFpEF. The study noted some trend towards a benefit in hospitalizations with ACEI and ARB in patients with HFpEF, but no statistical significance was established [21]. This is consistent with the findings of our study. The possible reason behind this could be the mechanism by which ACEI works on heart failure. ACEI ultimately decreases the cardiac muscle remodeling and halts the progression of myocytes fibrosis [23]. The cardiac remodeling and fibrosis are the mechanism for HFrEF. Initially, HFpEF was assumed to have the same pathophysiology as HFrEF. However, understanding of HFpEF has evolved. HFpEF has a different molecular mechanism for cardiac hypertrophy. Obesity, systemic arterial hypertension, and diabetes are the comorbidities which incites systemic inflammatory state. The systemic inflammatory state subsequently induces oxidative stress in the coronary microvascular endothelium, which after a series of molecular changes ultimately leads the cardiac myocytes to become stiff and hypertrophied [24]. Therefore, we can hypothesize that ACEI would have less benefits with HFpEF compared to HFrEF as seen in our study. 

In addition to BBT and ACEI, ARB is used routinely in patients with HFrEF. This drug class is associated with decreased mortality and hospital readmission in HFrEF [25]. Unlike large studies, such as the Val-HEFT trial that established a significant reduction in morbidity and lower rate of hospital admissions in the HFrEF patients treated with valsartan, there is a lack of evidence in mortality and hospitalization benefits of ARB in patients with HFpEF [26]. Two large trials, nonetheless, stand out from various studies investigating the outcomes of ARB therapy in HFpEF: the CHARM-Preserved and I-PRESERVE trials. CHARM-preserved, a multi-center, randomized, controlled trial, aimed to evaluate the impact on mortality and hospital admission for patients with HFpEF on candesartan compared to placebo. The study found no significant difference in mortality between the two groups, but demonstrated a modest yet statistically significant difference in terms of hospital admission for patients with ejection fraction > 40% [27]. Similarly, I-PRESERVE trial evaluated irbesartan vs placebo in 4128 randomized patients and found no benefit of ARB therapy on mortality or hospital admission between the study groups [28]. Findings from our study correlate with that of I-PRESERVE trial. In our study, use of ARB in patients with HFpEF did not reduce 30-day hospital readmission. Some factors that might have contributed to our finding include early drug discontinuation, either secondary to non-compliance or side effects such as hyperkalemia or kidney injury in the patients that were not investigated. Secondly, the dose of ARB therapy was not assessed in our study. A comparison of lower versus higher doses of therapy could yield results that would favor an association in a decreased rate of readmission. 

In our study, loop diuretics had a statistically significant decreased rate of 30-days hospital readmission. More specifically, in our study, we found loop diuretics to be the sole factor associated with a lower risk of readmission in our institution in HFpEF patients. The findings can be justified in that dyspnea due to congestion is the most common manifestation of patients who present with HF leading to hospitalization. Clinical congestion upon discharge might lead patient right back in to the hospital, especially in the setting of a poor renal function [29,30]. Consequently, diuretics are the cornerstone of therapy in such patients and can potentially reduce hospital readmission. 

### Limitation

Several limitations must be considered when considering the results of our study. Firstly, this is a single-center, retrospective study with a small sample size. Multicollinearity is problematic in these results and could be addressed in a considerably larger study that would allow the analysis of groups without overlapping categorization. Secondly, compliance with the medications prescribed at the time of discharge was assessed using only using the pharmacy database to ensure prescriptions were filled by the patient. Patient self-reporting about medication adherence was not incorporated in the study. Thirdly, our study looked at 30-day hospital readmission based on discharge medications only and did not evaluate other confounding variables, including insurance status, prescription coverage, referral to cardiac rehabilitation, first outpatient follow-up from index hospitalization—factors that might play a role in hospital readmission. Further, our study also did not evaluate time to readmission during a longer-follow up period or all-cause mortality. Therefore, given the clear heterogeneous nature of HFpEF, future more extensive studies are needed to analyze the effects of loop diuretics as well as various combination HF medications to identify hospitalization and/or mortality benefit and long-term outcome of each available medical therapy. 

## 5. Conclusions 

Our data supports decreased 30-day readmission in patient with HFpEF with the use of loop diuretics. However, no significant association was demonstrated with other cardioprotective medication classes including ACEI, ARB, BB and aldosterone receptor antagonist. This study highlights the real-world implication of lack of guidelines and treatment challenges with HFpEF management. Multiple combination of cardioprotective medication was not able to prevent 30-day readmission in patients with HFpEF emphasizing the significance of limiting medications for symptom resolution followed by a conservative approach. Therefore, our data hints at the significance of close follow-up after hospital discharge, treatment of underlying risk factors that contribute to fluid retention, and appropriate prescription of diuretics at the time of discharge to prevent hospital readmission in patients with HFpEF. This approach will also help lower the cost of medical expenses overall.

## Figures and Tables

**Table 1:** Hospital admission characteristics of study population.

Parameter	Not Readmitted within 30-Days (n = 317)	Readmitted within 30-Days (n = 128)	*p*-Value
**Mean age**	69 ± 12	67 ± 12	0.250
**Female (%)**	184	86	0.393
**Echocardiographic data**			
**Left ventricular ejection fraction (LVEF)**	57 ± 9.7%	58 ± 9.9 %	0.185
**Pulmonary artery pressure**	44 ± 17.7 mmHg	42 ± 15.2 mmHg	0.399
**E/e’ ratio**	16.33 ± 8.2	15.14 ± 7.7	0.223
**Body Mass Index**	37.42 (15.48)	35.72 (12.56)	0.260
**Medical comorbidities (%)**			
**Myocardial infarction**	71	23	0.165
**Hypertension**	141	60	0.843
**Hyperlipidemia**	130	62	0.437
**Obstructive sleep apnea**	118	45	0.345
**Atrial Fibrillation**	109	41	0.330
**Diabetes without complication**	130	51	0.417
**Diabetes with complication**	129	54	0.755
**Chronic Renal disease**	148	66	0.823
**Chronic lung disease**	193	92	0.241
**Peripheral artery disease**	30	8	0.194
**Tobacco use**	62	21	0.270

**Table 2:** Individual discharge medication class and combination of various medication classes by readmission status in the two-study group of patients.

Medication Class	Not Readmitted within 30-Days (n = 317)	Readmitted within 30-Days (n = 128)	*p*-Value
Angiotensin-converting enzyme inhibitors (ACEI)	87 (27%)	28 (20%)	0.106
Angiotensin receptor blocker (ARB)	21 (7%)	8 (6%)	0.740
Beta blockers (BB)	214 (68%)	85 (62%)	0.22
Loop diuretics (Loop)	199 (63%)	69 (50%)	0.011
Aldosterone receptor antagonists (Spironolactone)	19 (6%)	9 (7%)	0.829
Loop + ACEI	61 (19%)	20 (15%)	0.223
Loop + ARB	18 (6%)	2 (1%)	0.043
Loop + BB	144 (45%)	49 (36%)	0.049
Loop + Spironolactone	17 (5%)	8 (6%)	0.852
Loop + BB + ACEI	49 (16%)	19 (14%)	0.642
Loop + BB + ARB	15 (5%)	2 (1 %)	0.90
Loop + ACEI + Spironolactone	7 (2%)	0 (0)	0.79
Loop + ARB + Spironolactone	1 (1%)	0 (0)	0.59
Loop + BB + Spironolactone	13 (4%)	4 (3%)	0.534
Loop + BB + ACEI + Spironolactone	6 (2%)	0 (0)	0.104
Loop + BB + ARB + Spironolactone	1 (1%)	0 (0)	0.509
